# Diffusion kurtosis imaging in patients with tissue-negative transient ischemic attack

**DOI:** 10.3389/fneur.2022.1052310

**Published:** 2022-11-07

**Authors:** Jia Zhou, Rui He, Xiaoyu Xu, Xiaoer Wei, Minghua Li, Feng Wang, Yuehua Li

**Affiliations:** ^1^Department of Radiology, Shanghai Sixth People's Hospital Affiliated to Shanghai Jiao Tong University School of Medicine, Shanghai, China; ^2^Department of Neurology, Shanghai Sixth People's Hospital Affiliated to Shanghai Jiao Tong University School of Medicine, Shanghai, China

**Keywords:** diffusion kurtosis imaging, diffusion-weighted imaging, transient ischemic attack, ischemic stroke, tissue-negative

## Abstract

Approximately 50–60% of patients with a clinical transient ischemic attack (TIA) do not have diffusion-weighted imaging (DWI) evidence of cerebral ischemia. The purpose of this study was to assess the added diagnostic value of diffusion kurtosis imaging (DKI) in the evaluation of patients with TIA who have normal DWI findings. From September 2014 to May 2017, a total of 179 consecutive patients with suspected TIA were eligible for enrollment in our study. The inclusion criteria were a confirmed diagnosis of TIA confirmed by a stroke neurologist, MRI (including DWI and DKI) within 24 h after symptom onset, no stroke history, and no DWI lesion. A follow-up DWI was performed to establish stroke recurrence within a period of 90 days. A total of 98 patients who had no lesions on the baseline DWI were included for data analysis. Of these 98 patients, 31 (31.6%) had positive findings on the initial DKI. In 29 of the 31 (93.5%) patients, the location of the abnormality observed on DKI was consistent with the clinical symptoms. During the 90-day follow-up period, 14 (14.3%) patients developed recurrent stroke. The prevalence of recurrent stroke was higher in the DKI-positive group than in the DKI-negative group (29.0% vs. 7.5%, *p* = *0.01*). A comparison between the patients with and without recurrent stroke showed that an abnormality on the baseline DKI was associated with stroke recurrence. Furthermore, 8 of the 9 stroke patients in the DKI-positive group developed a new ischemic lesion in the artery territory corresponding to the initial DKI abnormality. The new findings suggest the predictive value of DKI on the recurrence of stroke in the patients with TIA who have negative findings on conventional DWI.

## Introduction

Transient ischemic attack (TIA) is traditionally defined as a temporary episode of neurological dysfunction due to a brief interruption of the blood supply to a confined brain area (1, 2). The phenomenon of TIA is time-based as the symptoms completely resolve within 24 h. In contrast, in the new definition of TIA is “tissue-based,” as TIA is now defined as a cerebral ischemic episode without any evidence of infarction on conventional imaging examinations (3, 4). TIA is a medical emergency and can be considered as a prodrome of ischemic stroke, which can cause permanent neurological deficits (5, 6). Diffusion-weighted imaging (DWI), a reliable magnetic resonance imaging (MRI) technique to help confirm the diagnosis of ischemic diseases, plays a pivotal role in the evaluation of patients with clinical symptoms compatible with a TIA. In patients with TIA, an acute ischemic lesion on DWI is a strong predictor of subsequent stroke (7–11). However, it is estimated that 50–60% of patients with TIA show no DWI abnormality (12–14). Although stroke risk is higher in patients with tissue-positive DWI than in those with tissue-negative DWI, the latter population is nevertheless at some risk (11, 15, 16). If the degree or duration of transient ischemia is insufficient to cause tissue injury, it may fail to detect any evidence of positive findings on DWI, making the diagnosis of TIA difficult. In such cases, additional MRI techniques, such as diffusion tensor imaging (DTI), are needed to further differentiate TIA from other transient neurological conditions that mimic TIA (17–19).

As an extension of conventional diffusion-based MR technique, diffusion kurtosis imaging (DKI) is applied to measure the non-Gaussian diffusion process in both white matter and gray matter, and this technique can provide a better understanding of the microstructure in brain tissue (20–25). DKI has shown promise in studies on Alzheimer's disease (26), mild traumatic brain injury (27), cerebral glioma (28), attention deficit hyperactivity disorder (29), as well as ischemic stroke (30). However, the application of DKI in the diagnosis of TIA is rarely reported.

In this study, we used DKI combined with DWI to predict the recurrence of stroke in patients initially presenting with TIA. Our study focused on a subset of TIA patients in whom no abnormalities were detected on DWI performed shortly after symptom onset. We aimed to determine whether DKI could provide further diagnostic information and help to estimate the risk of recurrent stroke in this subset of patients.

## Materials and methods

### Participant selection and clinical assessments

From September 2014 to May 2017, consecutive patients with suspected TIA who attended our emergency department were eligible for enrollment in this study. The inclusion criteria were as follows: (1) clinical symptoms of TIA (transient neurologic deficits with a presumed vascular cause that completely resolved within 24 h) on admission that were confirmed by a stroke neurologist at discharge; (2) MRI examination performed within 24 h of symptom onset; (3) no stroke history; and (4) negative finding on DWI. A follow-up DWI was performed to establish stroke recurrence within a period of 90 days. The diagnosis of recurrent stroke was based on the presence of stroke-related symptoms for more than 24 h and evidence of a new ischemic lesion on follow-up DWI. Patients who failed to undergo the MRI scanning and/or follow up within the certain period were excluded from our study. Demographic data, clinical symptoms at the baseline, symptom duration, time from onset to MRI, lesion location, etiology of TIA subtypes, ABCD2 score, personal status (hypertension, hyperlipidemia, diabetes mellitus, smoking status), and stroke recurrence were documented for each patient.

### Data acquisition

All patients underwent MRI on a 3T MRI scanner (MAGNETOM Verio, Siemens Healthcare, Erlangen, Germany) with an eight channel head coil. The MRI protocol includes the following: axial fast-spin-echo T_1_-weighted imaging, axial fast-spin-echo T_2_-weighted imaging, axial flow-attenuated inversion recovery, DWI, and DKI. DWI and DKI scans were obtained using the single-shot echo planar imaging pulse sequence. The scanning parameters for DWI were as follows: field of view (FOV) = 230 mm × 230 mm, repetition time (TR)/echo time (TE) = 5,400/94 ms, matrix = 162 × 162, phase partial Fourier factor = 6/8, slice thickness = 6 mm, distance factor = 30%, and *b*-values = 0 and 1,000 s/mm^2^. DKI scans were acquired using 6 *b* values (0, 500, 1,000, 1,500, 2,000, and 2,500 s/mm^2^) along 30 gradient-encoding directions with the following parameters: FOV = 230 mm × 230 mm, TR/TE = 2,300/109 ms, matrix = 128 × 128, phase partial Fourier factor = 6/8, slice thickness = 2.5 mm, and distance factor = 30%.

### Data processing

Diffusion kurtosis imaging post-processing was performed using the Diffusion Kurtosis Estimator tool (http://www.nitrc.org/projects/dke), which diffusivity and kurtosis maps were generated from all DKI images. Before the calculation, motion correction through a six-parameter rigid-body transformation was applied to all DKI images (31). After the modification, the generated mean diffusivity (MD), fractional anisotropy (FA), axial and radial diffusion, and mean kurtosis (MK) maps were reformatted into DICOM images using *b* = 0 DKI DICOM tags in an in-house nii-to-DICOM converter code (MATLAB, The MathWorks Inc.). The presence of an abnormality on DWI and DKI was decided in consensus by two independent neuroradiologists with 8 and 10 years of experience respectively, who were blinded to the clinical details. Patients were regarded to have an abnormality on DKI (DKI-positive patients) if a traceable hyperintensity was present on mean kurtosis images as compared with the contralateral hemisphere in the same parametric map.

### Statistical analysis

SPSS version 19.0 (IBM, Armonk, NY, USA) was used for statistical analyses. Quantitative data were expressed as mean ± standard deviation (SD). Differences in the measured data were tested using one-way analysis of variance (age, time from symptom onset to MRI scan, symptom duration, and ABCD2 score) or the chi-square test (sex, clinical symptoms, etiological TIA subtypes, hypertension, hyperlipidemia, diabetes mellitus, cigarette smoking, and stroke recurrence). *p* < 0.05 was considered significant (two-tailed tests) for all tests.

## Results

A total of 179 consecutive patients were eligible for enrollment in this study. Of these, 38 patients were excluded due to a history of ischemic stroke (16 patients), the loss of follow-up (6 patients), MRI scanning more than 24 h after symptom onset (5 patients), and the presence of motion artifacts leading to the failure of MRI acquisition (11 patients). Another 43 patients were excluded because they had positive findings on the initial DWI examination. Ultimately, 98 patients with suspected TIA in whom no abnormalities were detected on the initial DWI examination underwent a follow-up DWI and were selected for the final analysis. The mean age of the patients was 61.25 ± 11.90 years, and 52 of the 98 patients were male. The mean time from symptom onset to MRI scan was 293.71 ± 92.50 min, and the mean duration of symptoms was 27.72 ± 14.83 min. The mean ABCD2 score was 4.12 ± 1.17.

Among the 98 patients with no lesions on DWI at the baseline, DKI identified an abnormality in 31 (31.6%) patients. In 29 of these 31 (93.5%) patients, the location of the abnormality on DKI was consistent with the clinical neurological deficit. One patient who presented with right arm and leg weakness had a lesion in the right middle cerebral artery territory on DKI. Another patient with left sensory disturbance had an abnormality in the left anterior cerebral artery territory on DKI. The sensitivity and specificity of DKI in the detection of TIA-related cerebral lesions were 64.3% [95% confidence interval (CI), 0.49–0.63] and 73.8% (95% CI, 0.87–0.95), respectively. Moreover, the prevalence of subsequent stroke was significantly higher in patients with abnormalities on the initial DKI examination (DKI-positive group) than in those without any abnormalities on the baseline DKI (DKI-negative group) (29.0% vs. 7.5%, *p* = 0.01). No significant differences were detected in other factors, such as age, gender, time from symptom onset to MRI scan, and clinical symptoms, between the DKI-positive and DKI-negative groups (Table 1).

**Table 1:** Demographic features of patients with/without initial DKI abnormalities.

	**All patients (*n* = 98)**	**DKI-positive (*n* = 31)**	**DKI-negative (*n* = 67)**	***p*-Value**
Age (years)	61.25 ± 11.90	62.71 ± 12.93	60.57 ± 10.49	0.298
Gender (male)	52 (53.1)	15 (48.4)	37 (55.2)	0.264
Symptom duration (min)	27.72 ± 14.83	29.67 ± 17.44	26.82 ± 11.37	0.612
Time from symptom onset to MRI scan (min)	293.71 ± 92.50	284.36 ± 89.72	298.03 ± 94.08	0.536
**Vascular risk factor**
Hypertension	60 (61.2)	21 (67.7)	39 (58.2)	0.247
Diabetes	17 (17.3)	6 (19.4)	11 (16.4)	0.721
Hyperlipidemia	27 (27.6)	7 (22.6)	20 (29.9)	0.454
Smoking	31 (31.7)	9 (29.0)	22 (32.8)	0.707
**Etiological TIA subtypes**
Small vessel disease	16 (16.3)	5 (16.1)	11 (16.4)	0.971
Large artery atherosclerosis	36 (36.7)	12 (38.4)	24 (35.8)	0.783
Cardioembolic	20 (20.4)	5 (16.1)	15 (22.4)	0.475
Other determined	21 (21.4)	7 (22.6)	14 (20.9)	0.850
Undetermined	5 (5.1)	2 (6.5)	3 (4.5)	0.680
**TIA symptoms**
Motor weakness	73 (74.5)	26 (83.9)	47 (70.1)	0.147
Dysarthria	53 (54.1)	18 (58.1)	35 (52.2)	0.590
Aphasia	10 (10.2)	3 (9.7)	7 (8.2)	0.907
Sensory disturbance	36 (36.7)	13 (41.9)	23 (34.3)	0.468
Ataxia	9 (9.1)	2 (6.5)	7 (10.4)	0.524
Visual field defect	5 (5.1)	1 (3.2)	4 (6.0)	0.566
Diplopia	6 (6.1)	1 (3.2)	5 (7.5)	0.416
ABCD2 score	4.12 ± 1.17	4.35 ± 1.14	4.01 ± 1.19	0.564
90-day recurrent stroke	14 (14.3)	9 (29.0)	5 (7.5)	0.01

During the 90-day follow-up period, 14 (14.3%) patients developed recurrent ischemic stroke, while TIA or minor stroke were excluded. Of these 14 patients, 11 patients had a stroke within 7 days [relative risk (RR), 1.50; 95% CI, 0.95–2.38], and 3 had a stroke after 7 days and within 90 days (RR, 0.90; 95% CI, 0.45–1.79). And 9 of 14 had microstructural changes on the initial DKI, while 5 had no abnormalities on the initial DKI. The ABCD2 score did not significantly differ between patients with and without abnormalities detected by DKI. Positive DKI findings were associated with an increased short-term (within 7 days) and long-term (from 8 days to 90 days) risk of stroke recurrence. At 90 days, a multiple logistic regression model demonstrated that initial DKI-positive status [odds ratio (OR), 5.07; 95% CI, 1.53–16.78] is the only significant and independent factor in correlating with DWI abnormalities (Table 2). Furthermore, 8 of the 9 recurrent stroke patients in the DKI-positive group had new infarct lesions on the follow-up DWI in the cerebral artery territory corresponding to the initial DKI abnormality (Table 3).

**Table 2:** Demographic features of patients with/without recurrent stroke events within the 90-day follow-up.

	**Recurrent stroke (*n* = 14)**	**No recurrent stroke (*n* = 84)**	***p*-Value**
Age (years)	61.98 ± 12.73	61.13 ± 11.67	0.524
Gender (male)	6 (42.9)	46 (54.7)	0.409
Symptom duration (min)	28.41 ± 16.73	27.61 ± 12.28	0.819
Time from onset to MRI (min)	279.16 ± 89.17	296.14 ± 94.98	0.507
**Vascular risk factor**
Hypertension	10 (71.4)	50 (59.5)	0.397
Diabetes	3 (21.4)	14 (16.7)	0.663
Hyperlipidemia	5 (35.7)	22 (26.2)	0.460
Smoking	4 (28.6)	27 (32.1)	0.790
**Etiological TIA subtypes**
Small vessel disease	3 (21.4)	13 (15.5)	0.577
Large artery atherosclerosis	4 (28.6)	32 (38.1)	0.494
Cardioembolic	2 (14.2)	18 (21.4)	0.539
Other determined	4 (28.6)	17 (20.2)	0.482
Undetermined	1 (7.1)	4 (4.8)	0.708
**TIA symptoms**
Motor weakness	11 (78.6)	62 (73.8)	0.705
Dysarthria	8 (57.1)	45 (53.6)	0.804
Aphasia	3 (21.4)	7 (8.3)	0.134
Sensory disturbance	5 (35.7)	31 (36.9)	0.932
Ataxia	1 (7.1)	8 (9.5)	0.775
Visual field defect	2 (14.3)	3 (3.6)	0.092
Diplopia	2 (14.3)	4 (4.8)	0.169
ABCD2 score	4.47 ± 1.18	4.06 ± 1.17	0.147
Initial DKI-positive	9 (64.3)	22 (26.2)	0.005

**Table 3:** Characteristics of the nine recurrent stroke patients with initial DKI-positive findings.

**No**.	**Age (years)**	**Gender**	**Vascular territory of abnormality on initial DKI**	**Recurrent intervals (days)**	**Vascular territory of ischemic lesion on follow-up DWI**
1	72	M	L-MCA	2	L-MCA
2	65	M	L-MCA	9	L-MCA
3	70	F	L-ACA	1	L-ACA
4	89	M	R-MCA	3	R-MCA
5	53	F	L-PCA	8	L-MCA
6	78	M	R-MCA	7	R-MCA
7	51	F	L-MCA	4	L-MCA
8	43	F	R-MCA	10	R-MCA
9	87	M	L-MCA	3	L-MCA

L, left; R, right; M, male; F, female; ACA, anterior cerebral artery; MCA, middle cerebral artery; PCA, posterior cerebral artery.

## Discussion

To the best of our knowledge, this is the first study designed to determine the usefulness of DKI in the evaluation of patients with TIA who have no visible abnormality on conventional DWI. The present study indicates that DKI can provide additional information for the diagnosis of tissue-negative TIA in clinical practice. Even when no evidence of abnormalities is shown on DWI, subtle changes can be identified on DKI in some patients with TIA. Moreover, the initial DKI abnormality was associated with a higher risk of recurrent stroke within the 90-day follow-up period. Furthermore, we found that the new ischemic lesions on the follow-up DWI corresponded to the cerebral artery territory of the initial DKI abnormality.

As an extension of DWI, DKI has emerged as a neuroimaging technique with both improved sensitivity and specificity for the diagnosis and prognostication of ischemic stroke (24). Hui et al. (30) found heterogeneous kurtosis imaging characteristics in ischemic tissue that were not evident on conventional apparent diffusion coefficient (ADC) map. Zhang et al. (32) used DKI in comparison with DWI to evaluate ischemic lesions in a rat stroke model with middle cerebral artery occlusion, and concluded that DKI is more sensitive in detecting information about the microenvironment in the ischemic area. In this study, DKI detected microstructural changes in 31 patients with DWI-negative TIA. In addition, in almost all of these patients (29/31), the location of the abnormality on the initial DKI was consistent with the TIA-related neurological symptoms. DKI showed high sensitivity and specificity in the detection of cerebral lesions in our patient cohort. These findings demonstrate that DKI is a feasible and highly sensitive technique that can improve our understanding of the pathophysiological changes in patients with a clinical diagnosis of TIA. In ischemic tissue, the transient disruption of the sodium–potassium pump gets corrected after reperfusion, resulting in the absence of any changes in the intracellular or extracellular space. However, although the function of the sodium–potassium pump is restored, cellular destruction and mitochondrial swelling persist for a short while, and this may lead to an increase in the complexity or heterogeneity of the intracellular microenvironment. Therefore, the ischemic lesion may go undetected on the initial DWI. Besides the biological changes, technological such as resolution of MRI scanners, imaging parameters and magnetic susceptibility artifacts, may also contribute to hinder small DWI lesions (33). To better characterize the non-Gaussian water diffusion movement, the DKI scan uses both multiple b-values and multiple directions. In this study, we used 6 *b* values and 30 directions to enhance the reliability of the data. Furthermore, the DKI sequence is free of contrast agent injection and complicated post-processing, which makes it possible for this sequence to be widely applied in the clinical management of TIA.

The evidence of an abnormality on DWI is a reliable predictor of ischemic stroke following a TIA (7–11). In our study, we focused on the risk of recurrent stroke in a subset of TIA patients who had no lesions on DWI, and found that 14.3% of our patients developed stroke within 90 days of the TIA. The prevalence of subsequent stroke of DWI-negative TIA differed in various references. As reported, recurrent stroke occurred in ~3% of DWI-negative TIA patients within 7 days, and 9.5% within 5 years (34, 35). While multimodal MRI was applied, especially perfusion-weighted imaging (PWI), the incidence of recurrent stroke in DWI-negative TIA patients was relatively higher (36, 37). In our research, the prevalence of recurrent stroke was higher in the DKI-positive group than in the DKI-negative group. Moreover, comparison between those with recurrent stroke and those without recurrent stroke showed that the initial DKI abnormality was associated with a follow-up DWI lesion. These findings indicate that DKI positivity can be regarded as a valuable predictor of stroke in TIA patients with negative DWI findings. We speculated that the presence of abnormality on the initial DKI may reflect the severity of the underlying pathological mechanisms, which may in turn be attributed to a state of chronic hypoperfusion (the regional cerebral blood flow exceeds the threshold for causing TIA clinical symptoms but is still below the normal level) that is associated with an increased risk of recurrent TIA or stroke. In spite of the clinical symptomatic relief subsequent to reperfusion, any condition that decreases cerebral perfusion may lead to severe ischemia (38). This further supports the additional diagnostic value of DKI for identifying TIA patients who are at a high risk of subsequent stroke and in need of aggressive prevention therapies.

**Figure 1:**
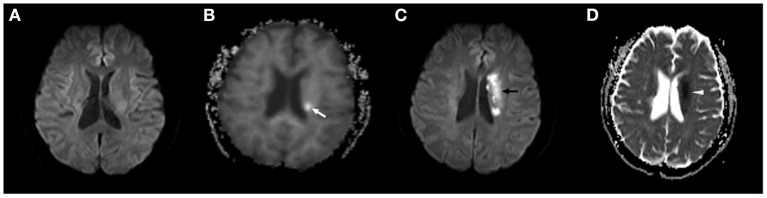
A 72-year-old male subject in the DKI-positive group developed a recurrent ischemic stroke 2 days after TIA onset. **(A)** No abnormality was found on the initial DWI scan. **(B)** A slightly hyperintense area in the left basal ganglia was observed on the baseline MK map (*white arrow*). **(C,D)** The new ischemic lesion is present in a similar location in the left middle cerebral artery territory on follow-up DWI (*black arrow*) and ADC maps (*white arrowhead*).

**Figure 2:**
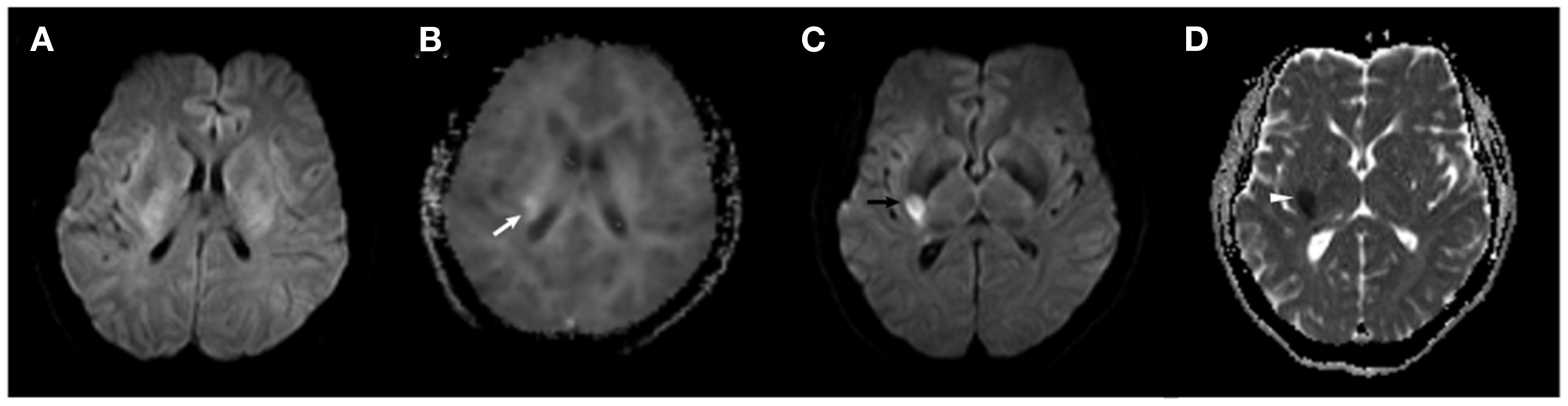
A 43-year-old female subject in the DKI-positive group developed a recurrent ischemic stroke 10 days after TIA onset. **(A)** No abnormality was found on the initial DWI scan. **(B)** A hyperintense area in the right basal ganglia was observed on the baseline MK map (*white arrow*). **(C,D)** The new ischemic lesion is present in a similar location in the right middle cerebral artery territory on follow-up DWI (*black arrow*) and ADC maps (*white arrowhead*).

In a subset analysis of nine recurrent stroke patients from the DKI-positive group, we found that in only one patient, the new lesion identified on DWI (in the right middle cerebral artery territory) did not match the initial DKI abnormality (in the left middle cerebral artery territory). In the remaining eight patients, the location of the lesion on the follow-up DWI corresponded to the artery territory of the initial DKI abnormality (Figures 1, 2), suggesting that the initial abnormality on DKI may potentially indicate the presence of the new ischemic lesion, but no correlation with the recurrent interval. It has been reported in a preclinical research that the lesion volume on MD was significantly larger than MK deficits, but decreased to approximately the size of MK and ADC after reperfusion, due to the heterogeneity of transient ischemia (39). However, in clinical TIA, the initial MK and recurrent ADC lesion differed in size, but corresponded to the location. According to previous reference, approximately 80% of stroke recurrence happened in the same territory of the index TIA at 7-day follow-up, which were basically consistent with our research (34). This new finding emphasizes the predictive value of DKI in the detection of microstructural changes in DWI-negative patients with a clinical diagnosis of TIA. The potential mechanism underlying this phenomenon is unclear. However, factors such as collateral circulation or pathological changes in the artery territory might be associated with recurrent ischemic stroke (34). Further large studies are necessary to elucidate this issue.

There are also several limitations to this study. First, our patients underwent only a 90-day follow-up, and their long-term outcomes were not evaluated. Second, 23.5% patients without positive findings on the initial DWI were excluded from the final analysis for various reasons, and this may have led to a selection bias. Third, we did not quantitatively measure the DKI-derived metrics in the ischemic brain tissue. Quantitative measurements of DKI-derived metrics in regions of DKI abnormality have been suggested, but this study was designed to preliminarily explore the usefulness of DKI in TIA. Therefore, these metrics were not measured in the present study. Whether a change in these metrics is correlated to symptom duration remains to be discovered. Finally, the follow-up MRI protocol was not consistent throughout the entire study. In our center, the follow-up DKI was interrupted in some patients with recurrent stroke in a certain time window who accepted intravenous thrombolysis therapy.

## Conclusion

This study indicates that DKI can provide additional diagnostic information on the cerebral microstructural changes in TIA patients who have negative findings on DWI. A study with a larger sample size is needed to confirm our results.

## Data availability statement

The original contributions presented in the study are included in the article/supplementary material, further inquiries can be directed to the corresponding author(s).

## Ethics statement

The studies involving human participants were reviewed and approved by Human Research Ethics Committee of The Sixth People's Hospital Affiliated to Shanghai Jiao Tong University School of Medicine. The patients/participants provided their written informed consent to participate in this study. Written informed consent was obtained from the individual(s) for the publication of any potentially identifiable images or data included in this article.

## Author contributions

JZ and XX performed the MRI scan and carried out the clinical data acquisition. RH and XW participated in the brain imaging data analysis. JZ and FW helped with the data interpretation and performed the statistical analysis. ML and YL conceived the study, participated in its design and coordination, and helped draft the manuscript. RH participated in the revision of the manuscript. All authors read and approved the final manuscript.

## Funding

This work was partly funded by the Shanghai Municipal Natural Science Foundation (No. 14ZR1432100), Shanghai Talent Development Fund (No. 201555), Shanghai Municipal Education Commission-Gaofeng Clinical Medicine Grant Support (No. 2016427), Clinical Science and Technology Innovation Project of Shanghai Shenkang Hospital Development Center (No. SHDC22015038), Shanghai Municipal Science and Technology Commission Medical Guide Project (No. 16411968900), and Shanghai Key Discipline of Medical Imaging (No. 2017ZZ02005). This work was also supported by the National Natural Science Foundation of China (Nos. 81471656 and 81671673).

## Conflict of interest

The authors declare that the research was conducted in the absence of any commercial or financial relationships that could be construed as a potential conflict of interest.

## Publisher's note

All claims expressed in this article are solely those of the authors and do not necessarily represent those of their affiliated organizations, or those of the publisher, the editors and the reviewers. Any product that may be evaluated in this article, or claim that may be made by its manufacturer, is not guaranteed or endorsed by the publisher.
